# Toric intraocular lens implantation – atypical cases

**DOI:** 10.22336/rjo.2020.67

**Published:** 2020

**Authors:** Alina Simona Lazăr, Bogdana Tăbăcaru, Simona Stanca, Tudor Horia Stanca

**Affiliations:** * “Prof. Dr. Agrippa Ionescu” Clinical Emergency Hospital, Bucharest, Romania; ** “Carol Davila” University of Medicine and Pharmacy, Bucharest, Romania

**Keywords:** toric intraocular lens, astigmatism, keratoconus, pellucid marginal degeneration, buphthalmos, congenital glaucoma, cataract, phacoemulsification

## Abstract

**Objective:** To describe the results of toric intraocular lens (IOL) implantation in three atypical cases (four eyes) with cataract and corneal astigmatism: one with bilateral keratoconus, one with pellucid marginal degeneration and one with buphthalmos due to congenital glaucoma.

**Methods:** Three patients (four eyes) with corneal astigmatism (one with bilateral keratoconus, one with pellucid marginal degeneration and one with buphthalmos due to congenital glaucoma) underwent cataract surgery by standard phacoemulsification and the implantation of toric IOLs in the capsular bag. The presence of corneal astigmatism was identified by automated keratometry and confirmed by Scheimpflug-based corneal tomography. The toric IOL implanted in all cases was a single-piece AcrySof Toric IOL (Alcon Laboratories, Inc.). Postoperative visual acuity, the reduction in the refractive astigmatism, the spherical equivalent (SE) and the rotational stability of the toric IOL were recorded for all the patients.

**Results:** Visual acuity increased and the refractive astigmatism decreased in all cases. In Case 1, the right eye achieved a postoperative uncorrected visual acuity (UCVA) of 20/ 20, a decrease in the refractive astigmatism from -3 DCyl to -0.75 DCyl and a spherical equivalent (SE) of -0.25. The left eye presented with a best-corrected visual acuity (BCVA) of 20/ 20, a decrease in the refractive astigmatism from -1.50 DCyl to -1.25 DCyl and a SE of -0.25. In Case 2, the postoperative UCVA was 20/ 20, with a decrease in the refractive astigmatism from -5.5 DCyl to -1 DCyl and a SE for the right eye of 0.00 D. In Case 3, the postoperative BCVA was 20/ 20, with a decrease in the refractive astigmatism from -4.75 DCyl to -1.50 DCyl and a SE of +1.25. No misalignment of the axis of the toric IOL was observed in any patient at subsequent follow-ups. The postoperative visual acuity was satisfactory for all the patients.

**Conclusions:** Toric intraocular lenses can be an effective option for implantation in patients with cataract and corneal astigmatism in atypical situations such as mild to moderate keratoconus, pellucid marginal degeneration and buphthalmos due to congenital glaucoma. Predicting the refractive outcome is difficult in atypical cases and the surgeon should have accuracy and consistency in the preoperative measurements, for achieving satisfactory postoperative results.

## Introduction

Toric intraocular lenses are designed to correct preoperative corneal astigmatism in modern cataract surgery. Since 1994, when Shimizu presented the first toric IOL, which was a non-foldable three-piece intraocular lens (IOL) made of poly-methyl methacrylate (PMMA) [**1**], many advances have been made in the toric IOL technology, which led to the improvement of the postoperative results and the overall patient satisfaction with the use of toric IOLs. 

In order to achieve satisfactory postoperative results, there are several factors to be taken into account when implanting a toric IOL, such as: the accurate measurement of the corneal astigmatism and the precise calculation of the IOL power, the accurate marking of the corneal meridians, the proper alignment of the IOL marks with the corneal marks, and the achievement of an accurate and stable postoperative IOL alignment. 

Even though the classical indication for toric IOL implantation is regular astigmatism, good results were also reported in cases of stable, mild central irregular astigmatism such as mild to moderate stable keratoconus [**2**], pellucid marginal degeneration [**3**] and post-corneal transplant [**4**].

Moreover, toric IOLs were inserted in phakic eyes with stable keratoconus, with good outcomes regarding ametropia correction [**5**,**6**].

Although there is no consensus regarding the use of toric intraocular lenses in atypical cases of patients with cataract and irregular corneas, the presence of corneal abnormalities is not regarded as absolute contraindication for toric IOL implantation, provided that a careful patient selection and consideration to several aspects regarding cataract surgery is taken into account. 

We presented the results of toric IOL implantation in 3 cases of 4 eyes of 3 different patients: one with mild to moderate bilateral keratoconus, one with pellucid marginal degeneration (PMD) and one with congenital glaucoma and buphthalmos. 

## Case report 

***Case 1***

**Fig. 1:**
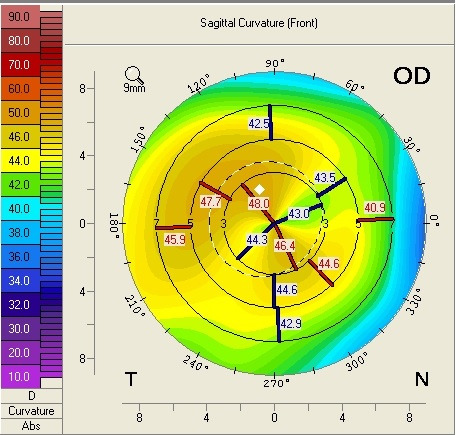
Scheimpflug Corneal Tomography Sagittal Curvature Map of the right eye showing oblique astigmatism with an “asymmetric bow tie” pattern (Case 1)

**Fig. 2:**
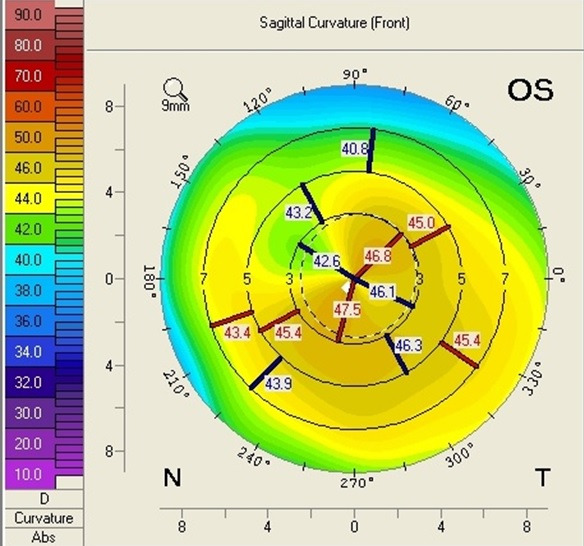
Scheimpflug Corneal Tomography Sagittal Curvature Map of the left eye showing irregular astigmatism and a pattern of “asymmetric bowtie with skewed radial axis” (Case 1)

A 62-year-old male presented to our ophthalmology clinic complaining of the decrease of visual acuity. Slit-lamp examination showed the presence of bilateral pseudoexfoliative material, bilateral nuclear cataract, more advanced in the right eye compared to the left eye, and asteroid hyalosis in the right eye. BCVA at presentation was 20/ 40 for the right eye, and 20/ 20 in the left eye. Refraction measured by automated refractometry was +1 DSf -3 DCyl 45° for the right eye, with a corneal astigmatism measured by automated keratometry of -2.5 DCyl 39° and keratometry (K) values for the steep and flat axis 47.50D at 129° and 45.00D at 39°. Refraction measured by automated refractometry was + 0.25 DSf – 1.50 DCyl 139° for the left eye with a corneal astigmatism of -1.50 DCyl 147° and keratometry (K) values for the steep and flat axis were 47.25D at 57° and 45.75D at 147°. Corneal assessment by means of Scheimpflug-based imaging revealed a very low central corneal thickness of 476µ for the right eye, and of 479µ for the left eye. The topographic evaluation of the right eye showed oblique astigmatism with an “asymmetric bowtie” pattern (**Fig. 1**), whereas the left eye presented with irregular astigmatism and a topographic pattern of “asymmetric bowtie with skewed radial axis” (**Fig. 2**). 

**Fig. 3:**
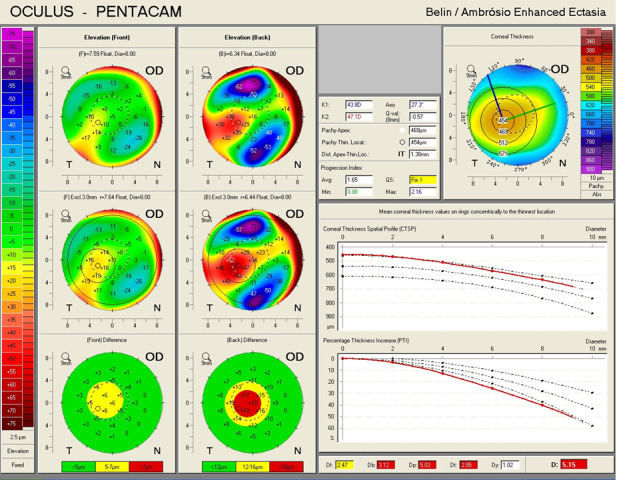
Belin/ Ambrosio Enhanced Ectasia analysis showing an ectatic aspect of the cornea in the right eye (Case 1)

**Fig. 4:**
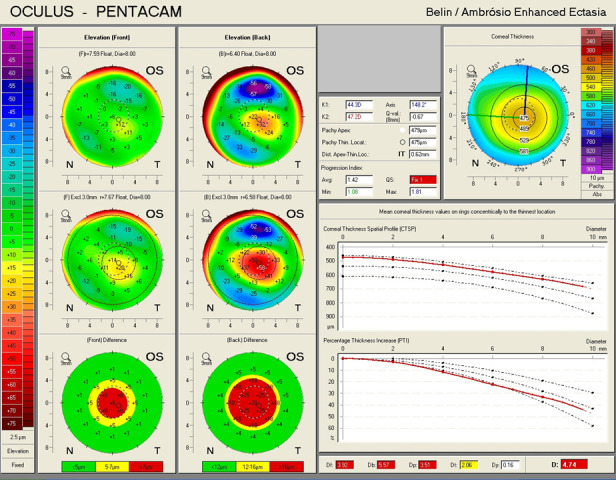
Belin/ Ambrosio Enhanced Ectasia analysis showing an ectatic aspect of the cornea in the left eye (Case 1)

An ectatic aspect of the cornea for both of the eyes was present, indicative of keratoconus (**Fig. 3**,**4**). 

The patient was kept under observation for a year, and the corneal topographic parameters remained unchanged. Cataract surgery was decided for the right eye and IOL power calculation was performed by means of both optical and ultrasonic biometry, for data consistency. Standard phacoemulsification was performed, with the implantation of a single-piece AcrySof Toric IOL (Alcon Laboratories, Inc.) of 17D in the capsular bag of the right eye, aligned at 128°. Postoperatively, the UCVA was 20/ 20, with a refraction for the right eye ROD: +0.00 DSf -0.75 DCyl 54°, a significant decrease in the refractive astigmatism from -3 DCyl to -0.75 DCyl and a spherical equivalent of SE: -0.25.

Three years later, the patient returned to our eye clinic for an ophthalmic consult. A significant decrease of visual acuity of 20/ 30 was noted in the left eye, due to cataract progression. No progression of corneal ectasia was noted in the left eye. The patient underwent cataract surgery for the left eye, standard phacoemulsification was performed and a single-piece AcrySof Toric IOL (Alcon Laboratories, Inc.) of 16.5D was implanted in the capsular bag of the left eye, aligned at 60°. Postoperatively, the BCVA was 20/ 20, with refraction for the left eye ROS: +0.50 DSf -1.25 DCyl 96°. There was an insignificant reduction of the refractive astigmatism from -1.50 DCyl to -1.25 DCyl for the left eye, but the postoperative spherical equivalent was SE: -0.25.

The patient was examined after a year postoperatively. No progression of keratoconus was observed, the lens did not rotate away from the desired axis, as confirmed by slit-lamp biomicroscopy and the patient reported satisfactory postoperative visual results. 

***Case 2***

A 59-year-old woman, known with the diagnosis of pellucid marginal degeneration (PMD), who had been stable for the last 2 years, presented to our eye clinic for ophthalmic evaluation, complaining of gradually decreased visual acuity in the right eye. The slit-lamp examination at presentation showed bilateral nuclear cataract. BCVA was 20/ 40 in the right eye, and 2/ 40 in the left eye. Refraction measured by automated refractometry was +5.25 DSf -5.5 DCyl 69° in the right eye and automated keratometry data showed corneal astigmatism of -4.5 DCyl 69°. Keratometry (K) values for the steep and flat axis were 44.75D at 159° and 40.25D at 69° in the right eye. The left eye could not be measured by means of automated refractometry. 

**Fig. 5:**
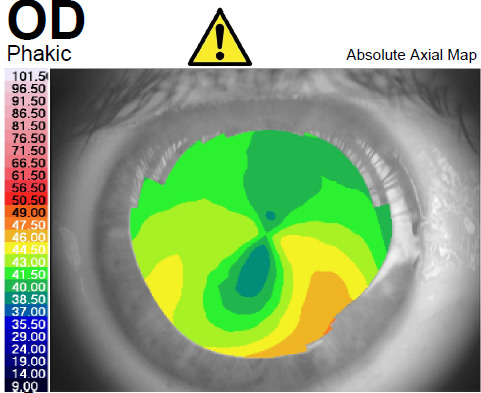
Corneal topography of the right eye showing an aspect of Pellucid Marginal Degeneration (Case 2)

**Fig. 6:**
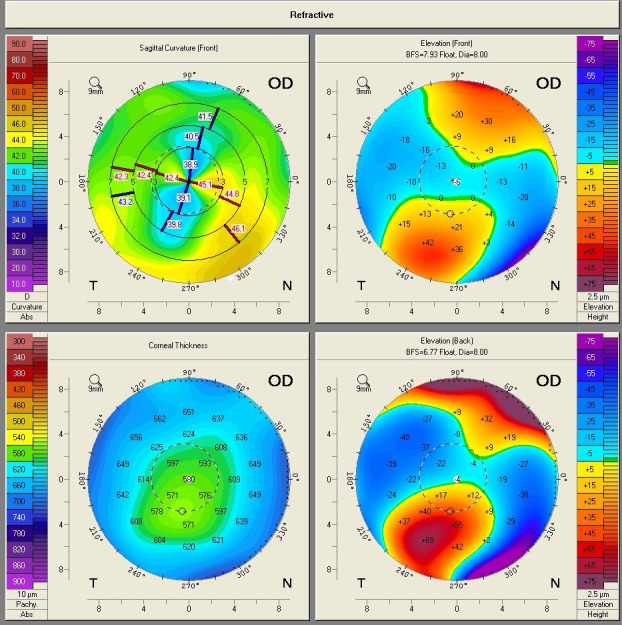
Scheimpflug Corneal Tomography of the right eye showing inferior steepening of the cornea (Case 2)

**Fig. 7:**
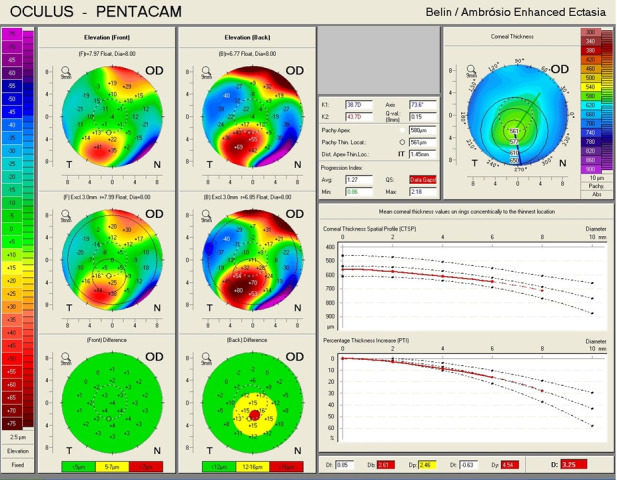
Belin/ Ambrosio Enhanced Ectasia analysis of the right eye showing ectatic changes in the right eye (Case 2)

**Fig. 8:**
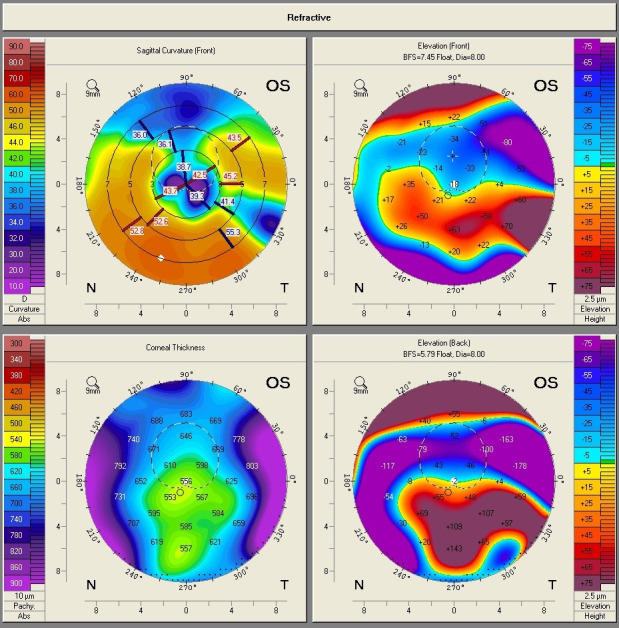
Scheimpflug Corneal Tomography of the left eye showing highly irregular astigmatism (Case 2)

**Fig. 9:**
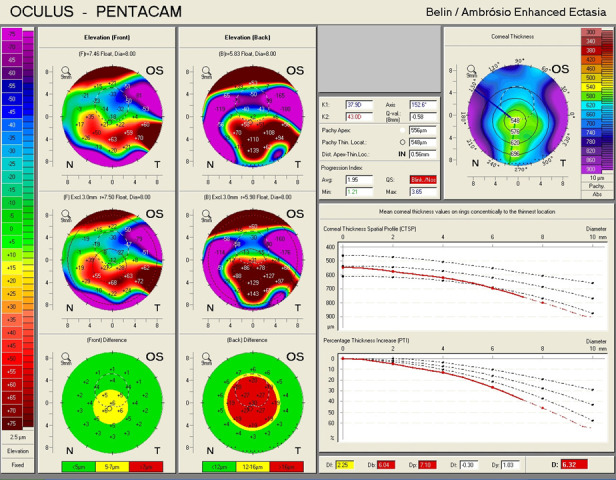
Belin/ Ambrosio Enhanced Ectasia analysis of the left eye (Case 2)

Corneal topography of the right eye showed an aspect of PMD, with peripheral inferior corneal steepening (**Fig. 5**). The aspect was illustrated also by Scheimpflug-based corneal tomography, which showed a symmetric and regular astigmatism in the central cornea of the right eye but an inferior steepening in the corneal periphery and an ectatic corneal aspect (**Fig. 6**,**7**). On the other hand, the left eye presented with a highly irregular astigmatism in the central cornea (**Fig. 8**,**9**). 

Cataract surgery was decided for the right eye. Optical biometry was performed. The patient’s right eye had an axial length < 22 mm; therefore, the Hoffer Q formula was used to calculate the IOL power, with a refractive target for emmetropia. Cataract surgery was performed with standard phacoemulsification technique, and a single-piece AcrySof Toric IOL (Alcon Laboratories, Inc.) of 26D aligned at 161° was implanted in the capsular bag. UCVA three months after surgery was 20/ 20 in the right eye, with a right eye postoperative refraction ROD: +0.50 DSf -1 DCyl 85°, a significant decrease in the refractive cylinder from -5.5 DCyl to -1 DCyl and a spherical equivalent for the right eye SE: 0.00 D. The toric IOL remained aligned at the desired axis and corneal topography remained unchanged at the three months follow-up. The postoperative visual result was satisfactory for the patient. 

***Case 3***

A 28-year-old male, with a history of congenital glaucoma and buphthalmos diagnosed at 3 months old, for which he underwent trabeculectomy and multiple iridectomies in both eyes, presented in our eye clinic with the complaint of decreased visual acuity in the right eye. Slit-lamp examination at presentation showed nuclear lens opacity in the right eye. BCVA was 20/ 50 in the right eye and UCVA was 20/ 20 in the left eye. Refraction measured by automated refractometry showed ROD: +1.25 DSf - 4.75 DCyl 2° in the right eye, with a corneal astigmatism measured by automated keratometry of -4.25 DCyl 4°, keratometry (K) values for the steep and flat axis were 45.00D at 94° and 40.75D at 4°. Refraction in the left eye was + 0.5 DSf - 0.5 DCyl 29°. 

**Fig. 10:**
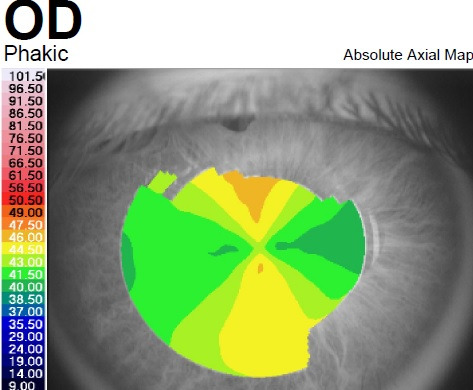
Corneal Topography of the Right Eye Showing regular corneal astigmatism with a “Symmetric bowtie” aspect (Case 3)

**Fig. 11:**
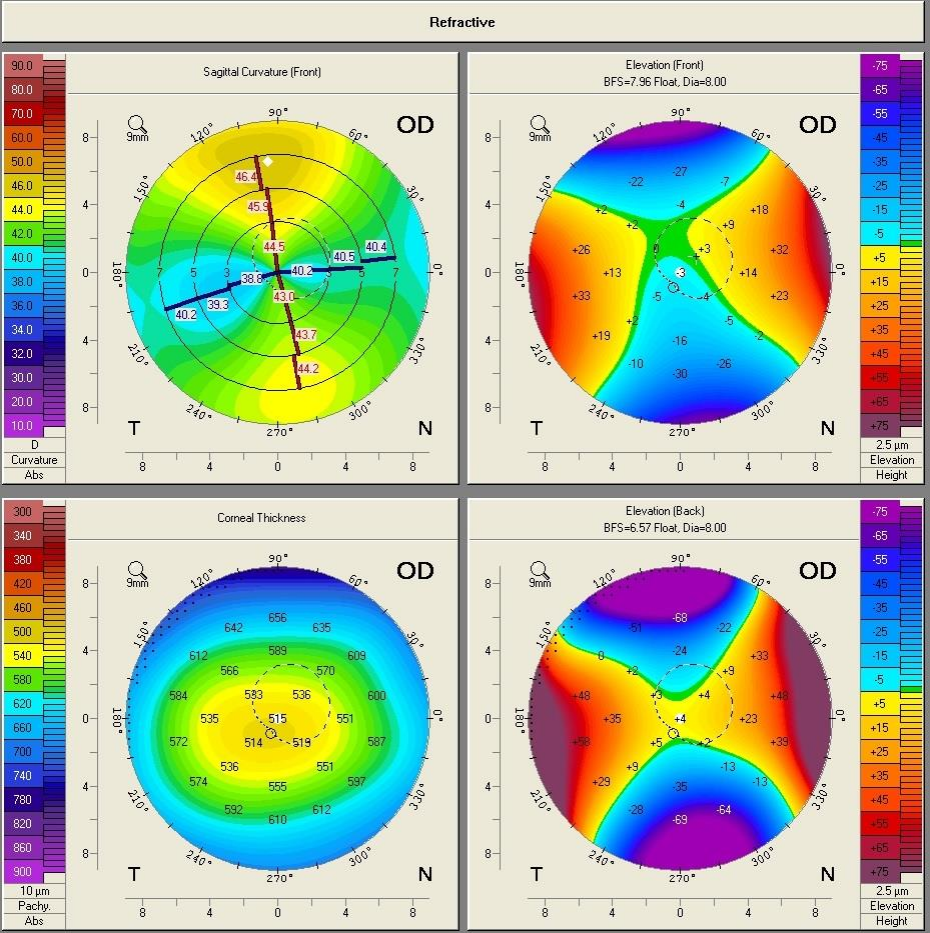
Scheimpflug Corneal Tomography of the right eye (Case 3)

**Fig. 12:**
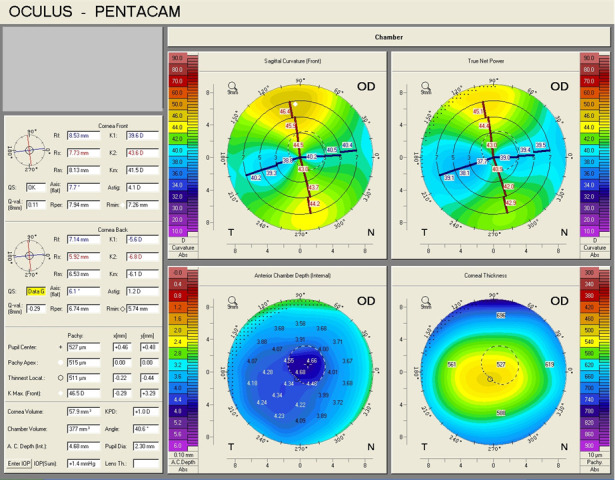
Scheimpflug Corneal Tomography of the right eye (Case 3)

Intraocular pressure (IOP) at presentation was 16 mmHg GAT in the right eye, and 17 mmHg GAT in the left eye. Placido-based corneal topography showed a highly regular corneal astigmatism in the right eye (**Fig. 10**). Keratometry measurement for flat K was 40.69D at 6° and for steep K was 45.17D at 96°, consistent with the one identified by means of automated keratometry. Biometric parameters measured by optical low-coherence interferometry showed a very high anterior chamber depth (ACD), of 5.24 mm, and a large white-to-white (WTW) corneal diameter of 13.16 mm. Scheimpflug corneal tomography confirmed the presence of regular astigmatism in the right eye (**Fig. 11**,**12**). 

**Fig. 13:**
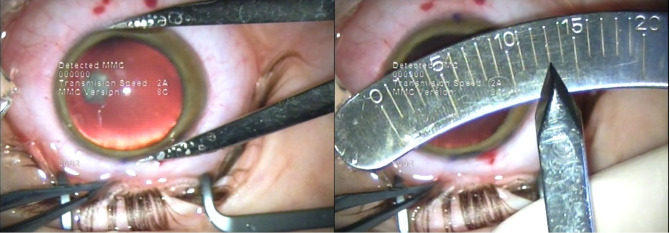
Right eye vertical corneal diameter of 13 mm as measured by calipers (Case 3)

**Fig. 14:**
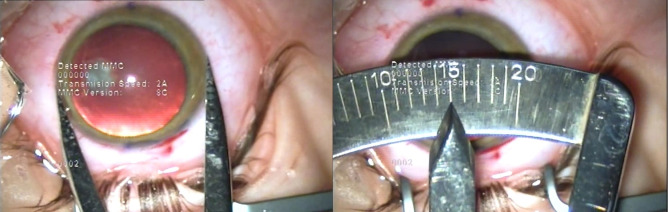
Right eye horizontal corneal diameter of 15 mm as measured by calipers (Case 3)

The axial length of the right eye measured by optical biometry was 25.02 mm. IOL power calculation was performed using the SRK/ T formula. It resulted that the optimal IOL for this patient was a toric IOL of 17.5D, with the axis aligned at 94°. Calipers were used to measure the vertical and horizontal diameter of the cornea, showing that the vertical corneal diameter (13 mm) (**Fig. 13**) was smaller than the horizontal corneal diameter (15 mm) (**Fig. 14**), making it possible to implant the toric IOL in the capsular bag. 

Uncomplicated cataract surgery with standard phacoemulsification was performed and a single-piece AcrySof Toric IOL (Alcon Laboratories, Inc.) of 17.5D, aligned at 94° was implanted. Postoperative BCVA 6 months after surgery was 20/ 20, with a refraction in the right eye ROD: +2.00 DSf -1.50 DCyl 20°, a decrease in the refractive astigmatism from -4.75 DCyl to -1.50 DCyl and a spherical equivalent SE: +1.25. Postoperative, the toric IOL showed good rotational stability and remained aligned at the desired axis at subsequent follow-ups. 

## Discussion

One of the main purposes of modern cataract surgery, apart from the replacement of the non-transparent natural crystalline lens with an optically clear implantable intraocular device, is to provide the patient a satisfactory postoperative visual result, attempting to target for spectacle independence after cataract surgery. Toric intraocular lenses (IOLs) are implantable intraocular devices designed to correct preoperative corneal astigmatism. They have been shown to provide better uncorrected distance visual acuity (UCDVA), greater spectacle independence, and lower amounts of residual astigmatism compared to non-toric IOLs, without showing an increased risk of complications [**7**].

Even though the general accepted indication for toric IOLs implantation is the presence of regular corneal astigmatism at baseline evaluation, careful patient selection and attentive consideration to several aspects of toric IOL implantation can provide good outcomes also in patients with atypical corneas. 

In this paper, we presented 3 different cases that underwent cataract surgery and toric IOL implantation, in eyes that did not fit into the classical category of indications for toric IOLs: one with bilateral mild to moderate stable keratoconus, one with pellucid marginal degeneration (PMD) and one with buphthalmos due to congenital glaucoma. 

In all the three cases, cataract surgery was performed by standard phacoemulsification technique under regional retrobulbar block, and toric IOLs were implanted in the capsular bag. 

Careful preoperative ocular examination was performed. The presence of corneal astigmatism was identified by automated keratorefractometry and confirmed by Scheimpflug-based corneal tomography, a method that investigates both the anterior and posterior surface of the cornea [**8**].

In the cases of corneal ectasia, the aspect of the corneal topography was an important criterion in the decision of toric IOL implantation. Even though, in general, patients with ectatic disorders are not regarded as good candidates for toric IOL implantation, there are several elements that play an important role in IOL selection for this category of patients. First of all, cases with a reasonable degree of symmetry and regularity of the astigmatism in the central 3 – to – 5 mm pupillary zone, which encompasses the visual axis, have the potential to achieve good postoperative outcomes with toric IOLs [**9**]. Corneal stability over time is a decision factor for toric IOL implantation in these patients. They need to present a history of stable topographic parameters at different subsequent follow-ups, with the absence of corneal ectasia progression in time, in order to be potential candidates for toric IOL implantation [**10**]. Moreover, the age is an important influencing element, after 50 years old the cornea achieving a high degree of stability and decreasing the likelihood of ectasia evolution [**11**].

In atypical cases of astigmatism, especially those associated with ectasia, preoperative planning is of significant importance. Consideration needs to be taken that, in these patients, the correction of a possible residual corneal astigmatism might be difficult to achieve. Means such as postoperative corneal laser ablative techniques, which, otherwise, have proven to be effective for astigmatism correction in patients without corneal pathologies [**12**-**14**], are not indicated in ectatic corneas [**15**].

Therefore, in unusual cases of astigmatism, biometry assessment is a significant challenge. Keratometry is one of the key parameters in IOL power calculation. In patients with corneal ectasia, however, keratometry presents great variability. A study that compared the repeatability of K readings between 5 devices, based on different principles of measurement (Scheimpflug pachymeter – Pentacam, Placido topographer – Eyesis, Scanning-slit corneal topographer – Orbscan, Partial coherence interferometry device – IOLMaster and Javal manual keratometer), concluded that the repeatability of K values in patients with keratoconus was good up to 55 D, whereas for K measurements above this value, all devices had low repeatability [**16**]. The lack of reliability regarding K values has an impact on IOL power calculations, and inaccurate IOL power measurement was reported in keratoconus eyes [**17**]. Moreover, in eyes with severe keratoconus, a large hyperopic shift may be encountered, due to an overestimation of the corneal power [**18**]. In a study that evaluated the refractive outcome of keratoconus patients who underwent cataract surgery with the implantation of a spherical IOL, biometry prediction error (BPE) was defined as the difference between the planned refraction determined by biometry and the spherical equivalent of the final refraction. In eyes with mild keratoconus (mean K < 48D), BPE was 0.0D. Patients with moderate keratoconus (mean K between 48D and 55D) presented with a mean BPE of +0.3D. Actual K values were used in all eyes with mild and moderate keratoconus. Patients in the severe keratoconus group (mean K > 55D) had a mean BPE of +6.8D in the category of patients in which the measured K values were used, and a mean BPE of +0.6D in the category of patients in which a standard K value of 43.25D was used [**18**]. A more posterior ELP [**19**], which may be due to the fact that eyes with keratoconus tend to have a deeper anterior chamber depth (ACD) [**20**] as well as longer axial lengths [**21**] may also contribute to the hyperopic shift. 

For measurement consistency and IOL power calculation precision, the preoperative keratometry in atypical eyes should be assessed by at least two different methods, for checking the measurement accuracy. 

IOL power calculation formula also constitutes a subject of debate regarding the best option for atypical cases of astigmatism. In mild keratoconus, SRK II formula was suggested to be more accurate in IOL power measurement, compared to other formulas, being the most reliable in all stages of keratoconus [**22**], but having less accuracy in severe keratoconus [**23**]. However, this is an older generation formula. Among the more modern formulas, the lowest refractive error was reported using SRK/ T [**24**], with keratometry derived from the 3-mm central zone in the axial map of corneal topography [**25**]. Recent studies are focused on newer generation formulas, in the attempt to find the one with the greatest accuracy in calculating the IOL power [**26**-**28**]. One study that evaluated the refractive accuracy of Hoffer Q, SRK/ T, Holladay I, Holladay II, Haigis and Barrett Universal II formulas in eyes with keratoconus, found that, while all formulas tended to have a hyperopic error, the Barrett Universal II formula was the most accurate for mild to moderate disease [**29**] but further research is needed in this regard. 

In the case of bilateral keratoconus presented in this paper, the IOL power calculation was made using the SRK/ T formula. The refractive target was emmetropia, and the spherical equivalent of the final refraction was 0.25D.

In the case of PMD, the IOL power calculation was made using the Hoffer Q formula, being the formula of election in eyes with axial length < 22 mm [**30**].

Apart from accurate pre-operative measurements and precise IOL power calculation, for a toric IOL to achieve its purposes of astigmatism correction, it needs to be properly aligned at the desired axis of implantation. Astigmatism correction is greatly influenced by the precise alignment of the toric IOL, as for each degree of toric IOL misalignment, 3.3% of the astigmatic correction is lost, and all the astigmatic correction is cancelled at 30° toric IOL position off the intended axis [**31**]. Regarding the image quality, the reduction of the quality of the image at 30-degree misalignment was reported to be less than 50%, whereas after 45 degrees of misalignment, the image quality was the same as if no toric correction existed [**32**]. A correct positioning of a toric IOL requires accurate preoperative determination and marking of the meridian of implantation and precise intraoperative alignment of the toric IOL at the desired axis. Several methods have been described for preoperative reference marking, such as manual methods, iris-fingerprinting techniques, image-guided methods and intraoperative aberrometry-based methods [**33**]. In the cases presented in this paper, preoperative marking of the cornea was made manually, at the slit-lamp, before the regional anesthesia was performed, using a needle and a surgical marking pen. The patients were in upright position, to avoid the cyclotorsion encountered in the supine position. To improve the patient comfort during marking, topical anesthesia with oxybuprocaine hydrochloride 4 mg/ ml was used. 

When it comes to postoperative outcomes, these are greatly influenced by the rotational stability of the toric IOL. The toric IOL implanted in all the four eyes was a single-piece AcrySof Toric IOL (Alcon Laboratories, Inc.), which proved excellent rotational stability in different studies [**34**], as well as in the three cases presented in this paper. Rotational instability after toric IOL implantation may happen due to several factors, such as: longer axial lengths [**35**,**36**], incomplete removal of ophthalmic viscosurgical device at the end of the case and early postoperative intraocular pressure fluctuations [**37**], the size of capsulorhexis [**38**], the IOL design and material [**37**] as well as ocular trauma, especially those causing significant leakage from the incision [**39**]. 

The horizontal corneal white-to-white (WTW) diameter is an important parameter for choosing the size of the IOL to be implanted. It is also one element to be taken into account regarding postoperative rotational stability of the IOL. There are contradictory results regarding the correlation between the horizontal corneal WTW diameter and the lens diameter, with different studies showing opposite results [**40**,**41**], but there is the presumption that an eye with increased anterior segment measurements is prone to postoperative rotation of the IOL due to an also large dimension of the capsular bag. An increased axial length was also linked to the capsular bag diameter [**36**,**42**]. 

This aspect was of great importance in the decision of toric IOL implantation in Case 3, which presented with a horizontal corneal WTW diameter of 13.16 mm as measured by optical low-coherence interferometry and 15 mm as measured by calipers. However, the patient had a vertical corneal diameter of 13 mm as measured by calipers. Even though the WTW measurements are significantly different depending on the instrument of measurement, as it is illustrated by different studies [**43**-**45**], in Case 3 they were larger than the average WTW measurement of a normal cornea, no matter the instrument used. Given the fact that the calculated axis was at 94°, the toric IOL, with a total diameter of 13 mm, could be implanted in the capsular bag, fitting the vertical diameter of the capsular bag and achieving postoperative rotational stability. The refractive postoperative outcome was a significant reduction in the refractive astigmatism. The final hyperopic shift noticed in the postoperative spherical equivalent in Case 3 could be due to the large corneal diameter and anterior chamber depth (ACD), which possibly had an impact on the accurate IOL power calculation. In the case of buphthalmos, the depth of anterior chamber is larger, due to the bulging of the cornea and flattening of the crystalline lens. It was suggested that in the eyes with large corneal diameters and deep anterior chambers, formulas that take into consideration WTW measurements as well as ACD should be considered for IOL power calculation, in order to achieve an optimal refractive outcome [**46**]. Moreover, to avoid a hyperopic shift, a more myopic refractive target should be taken into account [**46**].

In all the cases presented in this paper, a reduction in the refractive astigmatism after toric IOL implantation was registered. In Case 1, a less significant reduction of the refractive astigmatism was achieved for the left eye, probably due to the topographic pattern of the corneal astigmatism, which presented as an „asymmetric bowtie with skewed radial axis”. 

Postoperative visual acuity was satisfactory for all the patients. However, in atypical cases, patients with high expectations regarding spectacle independence after cataract surgery are probably not the best candidates for toric IOL implantation. In these cases, preoperative patient counseling is necessary and warning should be made regarding the possibility of incomplete astigmatism correction and the necessity of continuous spectacles wearing for the correction of the postoperative residual refractive error. 

## Conclusion

Toric IOLs could represent a suitable option for corneal astigmatism correction after cataract surgery even in atypical situations, such as mild to moderate keratoconus, pellucid marginal degeneration and congenital glaucoma with buphthalmos. A satisfactory postoperative result depends on careful patient counseling and examination, accurate pre-operative measurements, precise IOL power calculations, toric IOL alignment and postoperative IOL rotational stability. 

**Conflict of Interest**

The authors state no conflict of interest. 

**Informed Consent**

Informed consent has been obtained from all individuals included in this study.

**Authorization for the use of human subjects**

The research related to human use complies with all the relevant national regulations, institutional policies, is in accordance with the tenets of the Helsinki Declaration, and has been approved by the Ethical Committee of “Prof. Dr. Agrippa Ionescu” Clinical Emergency Hospital.

**Acknowledgements**

None.

**Sources of Funding**

None.

**Disclosures**

None.
